# Severe Life‐Threatening Hypokalemia Primarily Presented With Isolated Paralysis: Case Series From Ethiopia

**DOI:** 10.1002/ccr3.70062

**Published:** 2025-01-06

**Authors:** Getasew Kassaw Alemu, Seid Arage Asfaw, Lisanne Seifu Asres, Beniam Yohannes Kassa

**Affiliations:** ^1^ Nephrology Unit, Department of Internal Medicine, School of Health Sciences Addis Ababa University Addis Ababa Ethiopia; ^2^ Fetomaternal Unit, Department of Obstetrics and Gynecology Unit, School of Health Sciences Addis Ababa University Addis Ababa Ethiopia

**Keywords:** hypokalemia, hypokalemia‐induced paralysis, nonperiodic paralysis, pregnancy

## Abstract

Severe hypokalemia can primarily present as a weakness of the limbs, without any other clinical manifestation. A life‐threatening level of decreased serum potassium level can be unusually present with isolated weakness of the limbs and might be misdiagnosed, or the diagnosis may be delayed.

**Abbreviations**
ANC: antenatal care
EKG: electrocardiography
EMG: electromyography
NCT: nerve conduction test

## 1 Introduction

Hypokalemia is defined as a serum potassium level < 3.5 mEq/L and severe hypokalemia when it is < 2.5 mEq/L. Poor dietary intake, intracellular shift, and wasting from the body are the main causes of hypokalemia [1]. Patients may have a wide‐ranging spectrum of clinical presentations affecting the heart, muscle, gastrointestinal tract, central nervous system, and the respiratory system being the major ones [2]. Life‐threatening muscle weakness may also be a chief presenting symptom [3]. Hypokalemic periodic paralysis and hypokalemia‐induced nonperiodic paralysis could occur, the former being cyclical following some triggering factors while the latter one does not have this characteristic but follows hypokalemia. Patient evaluation encompasses a high index of suspicion, early detection, and prompt correction of potassium deficit with treatment of the underlying cause [4]. We present a series of three patients below.

## 2 Case 1

### 2.1 History and Examination

A 20‐year‐old male patient presented with three episodes of diarrhea and two episodes of vomiting in 2 days. The diarrhea was semi‐liquid content, and the vomiting was ingested matter with no fever or abdominal pain. Two days after the above symptoms started, he started to have weakness involving all his extremities and progressing over hours to the extent he failed to move. He had a history of dyspeptic symptoms (nausea and burning epigastric pain) for 2 years for which *H. pylori* eradication therapy was given after an endoscopy was done 1 year back that showed deformed bulb with a healing ulcer and inflamed surrounding mucosa on the duodenum and follicular‐appearing mucosa on the antral part of the stomach. Otherwise, there was no report of similar previous symptoms, medical comorbidity, medication, or toxin exposure. On physical examination generally, he was acutely sick‐looking with stable vital signs. Systemic physical examination was unremarkable except for mild epigastric tenderness and neurologic examination; the power of all his extremities was 2/5, and deep tender reflex was 2 with down‐going plantar reflex, normal tone, and sensory examination. He was conscious and oriented with negative meningeal signs.

### 2.2 Investigation and Treatment

**FIGURE 1:**
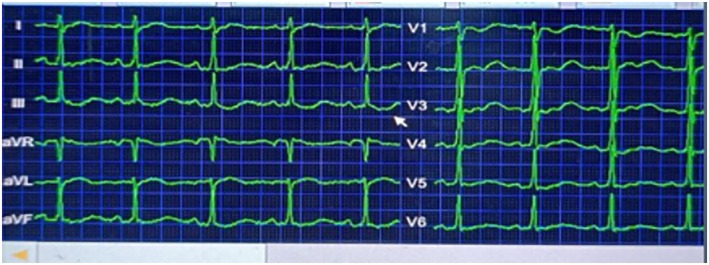
Electrocardiogram (ECG) showing sinus rhythm.

Laboratory investigations report: See (Table 1 and Figure 1). After admission to the hospital, he was resuscitated with normal saline over 4 h. After the lab result was available, he was given potassium chloride infusion of 60 mEq/L in 1000 mL normal saline over 6 h three times per day. The diarrhea was subsided by the second day of admission. After 240 mEq/L of potassium chloride was given, his lower extremity weakness was improved to a power of 3–4, and his serum potassium was 2.6. On the third day of the same potassium chloride dose, his serum potassium was 3.1, and he was able to walk to the toilet.

**TABLE 1:** Investigation summary of case 1.

Investigation	Value (on day 1 and serial values for some tests)	Reference
White blood cell	5300	4500–11,000/μL
Hemoglobin	9.3	♀: 12.6–16.2 and ♂: 13.1–16.7 g/dL
Platelet	259,000	150,000—450,000/μL
Creatinine	1.12–1.13–0.98 (days 1–3)	0.7–1.3 (mg/dL)
Urea	30–31–28 (days 1–3)	12–24 (mg/dL)
Urinalysis	Unremarkable	
Sodium	132	135–145 (mEq/L)
Chlorine	101	98–107 (mEq/L)
Potassium	1.7–2.6–3.1–3.6–3.9–4.3 (days 1–4)	3.5–5.2 (mEq/L)
Calcium	8.3	8.4–10.4 (mg/dL)
Phosphorus	3.6	2.8–4.6 (mEq/L)
Magnesium	2.0 mg/dL	1.3–2.1 (mg/dL)
Amylase and lipase	Normal for the laboratory reference range	
Coagulation panel (PT, PTT, INR)	Normal for the laboratory reference range	
Aspartate and alanine aminotransferase	Normal for the laboratory reference range	
Abdominal ultrasound	No study	
Nerve conduction test	Normal study	
Electrocardiography	Normal	

Abbreviations: INR, international normalized ratio; PT, prothrombin time; PTT, partial prothrombin time.

### 2.3 Outcome and Follow‐Up

On the day of discharge (i.e., the 4th day of admission), his potassium was 3.6, and walking without any difficulty. He was seen at the outpatient clinic twice, 3 days apart; he has no complaint at all; his serum K^+^ was 3.9, and renal function was normal. He was linked to the gastrointestinal unit for evaluation of the dyspepsia and discharged from renal follow‐up. Apart from investigations shown in Table 1 and potassium chloride treatment, further workup and therapy were not required.

## 3 Case 2

### 3.1 History and Examination

A 22‐year‐old primigravida mother who claims to be amenorrheic for the last 4 months presented with all‐extremity weakness of 1‐day duration. The weakness has started at all extremities simultaneously. She had intermittent nausea and vomiting of ingested matter for the past 2 months. She also had fatigue, nausea, and poor appetite for the same duration. Otherwise, there was no other complaint, similar episodes in the past, medication intake, or underlying comorbidity. On physical examination, upon presentation, generally, she was acutely sick‐looking with stable vital signs. On abdominal examination, there was a 14‐week size gravid uterus with prominent linea nigera, and her Glasgow coma scale (GCS) was 15/15, oriented with no cranial nerve and sensory examination abnormality. The strength of her extremities was 2/5 in all the extremities with deep tendon reflux of 1/4 at the knee and ankle and 2/4 on the remaining sites with downgoing plantar reflex. Other systems had normal physical examinations.

### 3.2 Investigation and Treatment

Laboratory tests done for her are summarized in (Table 2), and she was diagnosed with quadriparesis due to severe hypokalemia. After hospitalization, she was immediately started on management and the improvement was so rewarding.

**TABLE 2:** Investigation summary of case 2.

Laboratory	Value
White blood cell	65,000
Hemoglobin	10.1
Platelet	167,000
Urinalysis	Protein +2, blood +2, many RBC
Creatinine	1.44–1.16 mg/dL
Urea	24–22 mg/dL
Sodium	132 mEq/L
Chlorine	106.7 mEq/L
Potassium	1.69 mEq/L
Magnesium	2.3 mEq/L
Abdominal ultrasound, Electrocardiography	Normal finding for except 2nd trimester viable pregnancy
HCV, HBV, HIV, VDRL serology	Negative
Nerve conduction test	Not done

Abbreviations: HBV, hepatitis B virus; HCV, hepatitis C virus; HIV, human immunodeficiency virus; VDRL, venereal disease research laboratory.

She was given potassium chloride as a drip (40 mEq in 1000 mL normal saline three times per day, every 6 h); her potassium was 2.56 and 3.34 on the second and third admission dates.

### 3.3 Outcome and Follow‐Up

The weakness improved completely on the third day, and the patient went home by herself. There has been no vomiting or nausea since admission. She was given an appointment; she did not come since then.

## 4 Case 3

### 4.1 History and Examination

A 35 years‐old primigravida presented with complaints of nausea, vomiting, and burning epigastric pain for the last 3 months, and she was treated with ranitidine, domperidone, and graviscon (a combination of sodium bicarbonate, sodium alginate, and calcium carbonate) and folic acid during her Saudi Arabia follow‐up. She had a weight loss of 13 kg during this period (65–52 kg). Nausea, oral intake, and vomiting had improved with 1–2 episodes of vomiting in the past 1–2 months. Currently, she has easy fatigability, palpitation, and bilateral leg swelling with pain at the thigh bilaterally, with admission pulse rate and blood pressure being 97/min and 100/50 mmHg, respectively. Clinically her uterus was palpable (14 weeks in size).

### 4.2 Investigation and Treatment

Obstetric ultrasound showed a singleton live fetus consistent with the period of amenorrhea. Her spot urine ketone tested Positive (protein +2, ketone +2, leukocyte +2). Her complete blood count was normal a month back, and the current hemoglobin is—8.1 g/dL with mean corpuscular volume (MCV) of 76 fL, platelet—208,000/μL, and her hepatitis B surface antigen was positive with negative e antigen. The venereal disease research laboratory (VDRL) test, Liver enzymes, renal function test, widal titer, blood film, and brucella titer were negative. Deep vein thrombosis (DVT) was ruled out with a venous Doppler ultrasound of the legs. She was given therapeutic iron, cefixime, and navidoxine to be given in the outpatient setting. The next day, she developed weakness of the lower and upper limbs, which was severe enough to make her unable to come for the appointment. She had no history of headache or neck pain, trauma, psychiatric illness, diuretic or other related drug use, or previous similar episodes. On examination, the power of her upper limbs was 2/5, her lower limbs 0/5 with normal tone, but deep tendon reflexes were absent in the lower extremity. Cranial nerve function and bowel and bladder functions remained unaffected. Her serum potassium was 1.51 mEq/L with normal sodium and calcium. Her thyroid function tests (thyroid‐stimulating hormone [TSH] = 2.3 mIU/mL, fT4 = 1.2 ng/dL) were normal. She was diagnosed with hypokalemia‐induced paralysis and was initiated on potassium chloride therapy 40 mEq in 500 mL normal saline given as drip (8 hourly). Electrolyte monitoring was done every 12 h. Repeated obstetric ultrasound showed a single live fetus with the corresponding biometry at 15 weeks + 5 days. Potassium chloride replacement was continued for 48 h.

### 4.3 Outcome and Follow‐Up

The patient was walking with support on day 2 (serum potassium was 2.9 mEq/L) and without support by day 3 (serum potassium was 3.4 mEq/L). She was discharged with normal limb power with an appointment. Electrocardiography (EKG) was normal, but nerve conduction test (NCT) or electromyography (EMG) was not done.

## 5 Discussion

The cause of hypokalemia is multifactorial [5, 6, 7]. Vomiting and diarrhea play a major role as a cause of hypokalemia; what is peculiar in this study is that less frequent vomiting and diarrhea can lead to life‐threatening severe hypokalemia. These three patients had two important peculiarities. The first is that their presentation was severe, life‐threatening limb muscle weakness in the absence of other clinical hypokalemia evidence. The second peculiarity is that the cause of the hypokalemia was not as severe to both the patient to seek healthcare service before the current complaint and the physician to suspect hypokalemia as the cause of limb weakness. Hypokalemia was an incidental finding from routine laboratory tests in the three patients. There is limited evidence that hypokalemia can present with life‐threatening limb weakness except for periodic paralysis, which is more commonly observed. However, it is important to understand here from limited published evidence that this group of patients could present with not only less disturbing hypokalemia cause but also with life‐threatening weakness in the limbs, respiratory mechanics, or arrhythmia irrespective of the cause [8, 9]. It is also important to give due attention to the level of hypokalemia to causes muscle weakness. Three of the patients had serum potassium of < 2.0 mEq/L, and data from a recent study done in India has shown that serum potassium level < 2.0 mEq/L was associated with life‐threatening symptoms, of which all patients had quadriparesis, and in 10 patients the weakness was severe; six patients had respiratory failure, while one of these had required mechanical ventilation for 2 days. Unlike these patients, typical EKG changes were evidenced in seven of the patients in the study [10].

Therefore it is important to check routinely and as early as possible for hypokalemia in patients who present with acute muscle weakness so that treatment won't be delayed. Furthermore, the differentials for this kind of chief complaint may encompass a range of causes from structural to metabolic ones. Structural causes may include lesions in the brain, spinal cord, muscle, or peripheral nerves, and metabolic causes include vital organ dysfunction complications like kidney and liver failure and electrolyte imbalance. Routine proper neurologic and systemic evaluation is mandatory with timely investigation to reach on specific diagnosis. Severe hypokalemia is one of the medical emergencies where patients may present with life‐threatening periodic or nonperiodic body weakness either with or without fatal arrhythmia [11]. Unlike periodic paralysis, which likely recurs, nonperiodic hypokalemia‐induced paralysis is usually a one‐time event and is therefore unpredictable and can be due to potassium wasting, mandating determination of urinary potassium level and even arterial blood gas analysis [12].

It is also important to notice here that these patients presented clinically with sudden onset life‐threatening body weakness with normal electrocardiography (EKG) in the absence of any other explanation. There was no arrhythmic event with this degree of hypokalemia on 12‐lead EKG; patients were followed on monitors. This tells us that a patient's severe hypokalemia may present with muscle weakness in the absence of rhythm disturbance [10]. Hypokalemia, though is a possibility, was not given due attention initially until it was seen in the routine laboratory, as the presentation told us that the symptoms that would cause it weren't significant in these particular patients.

Urgent replacement of potassium, either orally or intravenously, is the cornerstone of therapy for hypokalemia despite the underlying cause [13]. Potassium deficit correction is a lifesaving treatment for fatal cardiac arrest and respiratory failure [14]. Early detection and correction of body potassium deficit is a life‐saving decision. Three of the patients had improved with potassium deficit correction and were discharged walking with an average length of hospital stay of 3.3 days (4, 3, and 3 days) and required an average potassium chloride of 540 mEq/L (720, 540, and 360 mEq/L) for the first, second, and third patient, respectively. The patients were followed with daily potassium laboratory and cardiac monitoring for possible fatal arrhythmia, as it is common [15].

It is important to notice in this study that two of the three patients were pregnant ladies; both of them had nausea and vomiting with severe hypokalemia complicated by muscle paralysis. Obstetricians and gynecologists, those who follow pregnant ladies in antenatal care, should be familiar that pregnant ladies with emesis gravidarum or hyperemesis gravidarum are risk to develop hypokalemia‐related severe muscle weakness, as hypokalemia is one of the commonest complications of hyperemesis gravidarum [16, 17].

This study may give evidence on less severe causes of hypokalemia leading to severe hypokalemia with severe body weakness and how early potassium replacement changes the clinical course. Though the monitoring was not as per the recommendation, none of the patients had iatrogenic hyperkalemia in this series.

The main strength of this study is that it tries to give evidence that hypokalemia‐induced paralysis could follow milder symptomatic causes. It also tells us routine serum potassium checkups are mandatory in patients with acute paralysis. The main limitation of this case series is that it lacks some basic investigations, including urinary potassium level, ABG, and nerve conduction tests that were not done for all.

## 6 Conclusion

Hypokalemia‐induced nonperiodic paralysis may cause severe muscle weakness, and it is important to have a high index of suspicion in patients who present with this complaint to detect them early, and prompt correction of potassium deficit had saved these three patients, per this study. pregnant mothers have a lower threshold for hypokalemia‐related complications related to emesis or hyperemesis gravidarum. Therefore, physicians working at ANC clinics should have a high index of suspicion to detect this life‐threatening yet easily treatable clinical condition early.

## Author Contributions

**Getasew Kassaw Alemu:** conceptualization, data curation, formal analysis, investigation, methodology, writing – original draft. **Seid Arage Asfaw:** data curation, formal analysis, supervision, validation. **Lisanne Seifu Asres:** investigation, supervision, validation, visualization. **Beniam Yohannes Kassa:** investigation, methodology, visualization, writing – review and editing.

## Ethics Statement

The authors have nothing to report.

## Consent

Written informed consent was obtained from the patients.

## Conflicts of Interest

The authors declare no conflicts of interest.

## Data Availability

All data sets on which the conclusion of the cases for this study are based are available as a medical record document and are available from the corresponding author on reasonable request from the editor.
